# Vancomycin-Resistant Entrococci colonization in chronic hemodialysis patients and its risk factors in southern Iran (2005-2006)

**Published:** 2012-10-30

**Authors:** S Shaghaghian, B Pourabbas, A Alborzi, M Askarian, J Mardaneh

**Affiliations:** 1 Community department of Shiraz University of Medical Sciences, Shiraz, Iran; 2 Professor Alborzi Clinical Microbiology Research Center, Shiraz University of Medical Sciences, Shiraz, Iran

**Keywords:** Vancomycin Resistant Entrococci, Hemodialysis, Risk Factor, Iran

## Abstract

**Background:**

Vancomycin-resistant enterococci (VRE) recently have emerged as a nosocomial pathogen among dialysis patients. This study aims to appraise the prevalence, incidence density and risk factors for VRE colonization among these patients.

**Methods:**

In this prospective study, 782 stool or rectal swab specimens were collected from 250 chronic hemodialysis patients with an interval of at least one month. To identify the risk factors of VRE colonization, demographic and health data of VRE+ and VRE- patients were compared.

**Results:**

VRE colonization was detected in 55 (22%) patients during study. Incidence density of one case per 79.6 patient-month of follow up was estimated.The only significant difference between the data collected from VRE+ and VRE- patients was observed in antibiotic consumption (P<0.001).

**Conclusion:**

VRE colonization is relatively high and rapidly spreading among chronic dialysis patients. It is strongly associated with recently antibiotic consumption.

## Introduction

The prevalence of antimicrobial-resistant microorganisms in various health care settings have increased dramatically in the last decade.(1) Certain groups of pathogens, in which the frequency of resistance has risen rapidly in recent years pose a particular threat to severely ill patients. The recent emergence of vancomycin-resistant entrococci (VRE) as a nosocomial pathogen is an outstanding example of this new danger to at risk patients.(2) Vancomycin resistant enterococci have been common in patients with end-stage renal disease (ESRD), who comprised 17% to 29% of VRE^+^ patients in three hospital-based studies.(3)

The increased risk of acquiring VRE in chronic hemodialysis (HD) patients is due to several factors including; extensive contacts with the healthcare system, close proximity to other VRE patients, multiple comorbid condition.(4)

In a study, functional statues, injection drug use, hospitalization and antimicrobial especially vancomycin receipt were risk factors for VRE colonization.(3) In another study, it was shown that the only statistically significant difference between VRE^+^ and VRE^-^ patients was in the volumes of previous exposures to intravenous (IV) vancomycin receipt and the risk factors for VRE colonization in nonuremic populations were not found in HD populations.(5)

The present study aims at investigating the prevalence, incidence density and risk factors for VRE colonization among chronic HD patients in two major HD centers in Shiraz, southern Iran.

## Materials and Methods

The study was performed in two HD units located in two major teaching hospitals in Shiraz (Namazi and Faghihi hospitals), affiliated with Shiraz University of Medical Sciences, Iran. They are specified for adults with 18 and 15 beds, respectively. 

Of the 352 HD patients, dialyzed during four daytime shifts at the out-patient dialysis unit during December 2005 to August 2006, nine months, 250 (71%) with informed consents enrolled in the present study. The acute cases (duration of HD<1 month) were excluded.

In this prospective study, the obtained rectal swabs or stool specimens with an interval of at least 1 month were cultured for VRE to determine the period prevalence and the incidence density of VRE colonization in the studied chronic HD patients. During the study period, 782 stool or rectal cultures according to patients’ preferences were performed on the patients. These patients were followed up 955.5 person-month.

The specimens were inoculated onto bile-esculin agar plates with and without vancomycin and into bile-esculin broth supplemented with it. Then they were subcultured. Isolates recovered from stool or rectal swabs were presumptively identified as enterococci by colonial morphology, Gram's stain, the absence of catalase production, the presence of pyrrolidonylarylamidase by hydrolysis of L-pyrrolidonil-b-naphthylamide (PYR test, Sigma Co.), tolerance to 6.5% sodium chloride. Species identification was carried out with a test scheme that is based on carbohydrate fermentation and arginine deamination. Carbohydrate fermentation tests were performed in heart infusion broth base with 1% mannitol, sorbitol, sorbose, arabinose, melibiose, sucrose, trehalose, lactose, glycerol, maltose (Sigma Co.). Arginine deamination was tested with Moeller arginine decarboxylase broth (Merck). Yellow pigmentation was observed after overnight growth on Müeller Hinton agar (Merck) supplemented with 5% sheep blood and by taking a sweep of the plate with a cotton swab. The pyruvate utilization test was observed with a broth containing 1% pyruvate. The susceptibility of vancomycin resistant isolates to other antibiotics [ampicillin (10µg), chloramphenicol (30µg), ciprofloxacin (5µg), erythromycin (15µg), imipenem (10µg), linozolide (30µg), penicillin (10µg), tetracycline (30µg), teicoplanin (30µg) and co-trimoxazole (1.25-23.75µg)] ( MAST Co. UK) was determined using a disk diffusion method (Kirby- bauer) on Mueller-Hinton agar plates (Merck) and interpreted according to the method of CLSI. 

Standardized forms were used to obtain data from clinical and administrative records or patients’ interviews were performed. A baseline form containing demographic and health data was filled at the initiation of the study for all the patients and follow up forms containing data on hospitalization, consumption of antibiotics, corticosteroid or other immunosuppressive drugs in the periods between the two specimen collection and hemoglobin, albumin and WBC count of the preceding month were completed on monthly visits. To identify potential risk factors, we compared data of VRE^+^ and VRE^-^ patients. VRE^+^ patients were defined as having at least one positive VRE culture during the study period. The data were analysed by using the following statistical tests in SPSS 11.5 software:

1. Independent Sample T Test for age, duration & frequency of dialysis, albumin, Hb and WBC count.

2. Chi Square test for sex, hospitalization, antibiotic consumption, underlying kidney disease, presence of other chronic disease and mobility status. 

3. Fisher’s Exact Tests for immunodeficiency status.

Variables with P<0.25 in univariate analysis were included in the logistic regression model (Enter method) with VRE colonization defined as dependant factor. For all analysis, a P<0.05 was considered significant. The ethics committee at the Clinical Microbiology Center reviewed and approved all the obtained informed verbal consents.

## Results

Of the 352 HD patients, 250(71%) participated in the study and their stool samples were cultured one to six times. The mean age was 52.5 years and 60% were male. The mean number of months undergoing HD was 27 (Table 1). During the study period, 782 stool or rectal swab specimens were obtained from 250 patients and were cultured for VRE, of which 55 (22%) were positive (period prevalence) in one of their cultures. Among samples with VRE (n=55), 14 (25.5%) were Enterococcus Faecium; 21 (38.1%), Enterococcus Casseliflavus; 16 (29.1%), Enterococcus Gallinarium; and 4 (7.3%), Enterococcus Faecalis.

**Table 1:** Comparison of VRE-positive and VRE-negative patients at Shiraz hemodialysis centers, (2005-2006) (N=250)

	Total (N=250)	VRE (N=55)	No VRE (N=195)	P-Value (Univariate Analysis)
Age_(years)_, Mean ± SD a	52.49 ± 15.85	50.59 ± 14.64	53 ± 16.16	0.88
Sex (%)				0.982
Male	150(60)	32 (58.2)	118 (60.5)	
Female	100(40)	23 (41.8)	77 (39.5)	
Underlying kidney disease (%)				0.08
Diabetes mellitus	77 (30.8)	8 (14.5)	69 (35.4)	
Hypertension	75 (30)	12 (21.8)	63 (32.3)	
Infection	16 (6.4)	3 (5.5)	13 (6.7)	
Glomerulonephritis	17 (6.8)	6 (10.9)	11 (5.6)	
Others b	65(26)	26 (47.3)	39 (20.0)	
Other chronic disease (%)				0.189
With other chronic disease	48 (19.2)	14 (25.5)	34 (17.4)	
Without other chronic disease	168 (67.2)	34 (61.8)	134 (68.7)	
Unknown b	34(13.6)	7(12.7)	27(13.9)	
Mobility status (%)				0.248
Normal	172(69)	41 (74.5)	131 (67.2)	
Limited	67(27)	14 (25.5)	53 (27.2)	
Bedridden	11(4)	0 (0)	11 (5.6)	
Duration of dialysis(Months), mean ± SD	27.2 ± 24.04	28.14 ± 21.69	26.94 ± 24.73	0.76
frequency of dialysis(Months), mean ± SD /Months	8.84 ± 2.77	8.64 ± 2.21	8.88 ± 2.87	0.57
Immunodefficiency (%)				0.981
With Immunodefficiency	18(7.2)	4 (7.3)	14 (7.2)	
Without Immunodefficiency	232(92.8)	51(92.7)	181(92.8)	
Hospitalization during the previous month (%)				0.459
With hospitalization	85(34)	21 (38.2)	64 (32.8)	
Without hospitalization	165(66)	34(61.8)	131(67.2)	
Antibiotic consumption during the previous month(%)				<0.001
With antibiotic consumption	101(40.4)	33 (60)	68 (34.9)	
Without antibiotic consumption	149(59.6)	22(40)	127(65.1)	
Albumin(mg/dL), mean ± SD	3.97 ± 0.61	3.96 ± 0.55	3.99 ± 0.58	0.814
Hb(mg/dL), mean ± SD	9.60 ± 2.06	9.56 ± 1.8	9.65 ± 1.9	0.773
WBC count, mean ± SD	6.17 ± 1.42	5.6 ± 1.43	6.2 ± 1.40	0.017

^a^SD = standard deviation

^b^These groups were not included in the statistical analysis

Of the 250 patients, 43 (17.2%) were colonized with VRE when initially cultured. The remaining patients received a total of 955.5 patient-months of follow-up and 12 incident cases of VRE colonization were detected over the course of the study resulting in an incidence density of one case per 79.6 patient-months of follow up.

Risk factors of VRE^+^ and VRE^-^ patients are compared in table 1. In univariate analysis, VRE colonization was not related to age, sex, duration and frequency of HD but significant differences between VRE^+^ and VRE^-^ patients were in antibiotic consumption in the previous month(P <0.001) and WBC count(P=0.017).

In logistic regression analysis in which VRE colonization was defined as dependant factor and WBC Count, underlying kidney disease, presence of other chronic disease, mobility status and consuming antibiotics were defined as independent factors; the only significant difference was in antibiotic consumption (P < 0.001). (Table 2)

**Table 2:** Multivariate analysis of risk factors associated with VRE colonization at Shiraz hemodialysis centers (2005-2006) (N=250)

Risk Factors	Wald	P Value	OR a (95% CI b)
Consuming antibiotics during the previous month	31.36	<0.001	54(13.37-218.16)
WBC Count	0.196	0.658	0.90(0.58-.41)
Underlying kidney disease	0.231	0.631	0.90(0.62-1.34)
Other chronic disease	0.429	0.513	1.74(0.33-9.23)
Mobility status	0.345	0.557	0.68 (0.19-2.48)

^a^OR=Odds Ratio

^b^CI= Confidence Interval

Because clinically important vancomycin resistance enterococci has been found most commonly in E. faecalis and E. faecium(1), antibiotic consumption in the previous month was compared between patients colonized with these two organisms and VRE^-^ patients. We also found significant difference between these two groups (66.7% of patients with vancomycin resistant E. faecalis and E. faecium vs. 34.9% of VRE^-^ patients, P=0.008).

For the isolates resistant to vancomycin, susceptibility to a variety of antibiotics was determined by disk diffusion method. The results of these antibiotic sensitivity tests are presented in table 3, according to which, sensitivity of VRE to these antibiotics ranges between 7.3% to 78.2% and these enterococci were most sensitive to imipenem, gentamycin and co-trimoxazol, sequentially.

**Table 3:** Antibiotic sensitivity patterns of VRE isolated from Shiraz hemodialysis patients (2005-2006)

Antibiotic	Sensitive (%)	Resistant (%)	Intermittent (%)
Imipenem	43 (78.2)	12 (21.8)	
Gentamicin	42 (76.4)	12 (21.8)	1 (1.8)
Co-trimoxazole	41 (74.5)	10 (18.2)	4 (7.3)
Penicillin	40 (72.7)	15 (27.3)	
Ampicillin	38 (69.1)	17 (30.9)	
Teicoplanin	35 (63.6)	14 (25.5)	6 (10.9)
Linozolide	29 (52.7)	6 (10.9)	20 (36.4)
Chloramphenicol	27 (49.1)	13 (23.6)	15 (27.3)
Tetracycline	22 (40)	28 (50.9)	5 (9.1)
Ciprofloxacin	6 (10.9)	36 (65.5)	13 (23.6)
Erythromycin	4 (7.3)	29 (52.7)	22 (40)

## Discussion

The present prospective study assessed the colonization with VRE in HD patients, and VRE prevalence, incidence density and risk factors among HD patients at two Shiraz dialysis centers were reported. Overall, 55(22%) of the 250 patients were VRE^+^.

Several studies to date have examined the prevalence of VRE in HD populations , frequencies ranging from 5.8% to 9.5% were demonstrated in four studies in USA.(3,5-7) In studies conducted in Ireland(8) and Brazil(9), VRE prevalence were 13% and 14.4% respectively. Lower percentages were reported from Israel (4.8%)(10) and Athens(3.9%)(11). 

VRE colonization in our study was relatively high compared to the findings reported from other studies. This high rate was partly attributed to the applied methodology, in which period prevalence of a longitudinal study for 9 month duration was reported whereas some previous studies were cross sectional and reported point prevalence(5, 9,11. For better comparison, we also decided to calculate a point prevalence, and in doing so, we evaluated specimens collected in December 2005 (first period of specimen collection) that showed 30 (19.6%) of 153 patients treated at these two HD units were VRE^+^. The resulting point prevalence (19.6%) is also higher compared to that in previous studies. Moreover, in a study conducted in the same HD centers of Namazi and Faghihi hospitals in March 2005, the point prevalence was 6.2% (12), which is less than 1/3 of that in the present study conducted 9 months later. 

This rapid spread of VRE colonization is comparable with previous studies. In a Greek study, the prevalence of VRE colonization was 1.2% in 1999 and 34.9% in 2003.(13) A CDC (Centers for Disease Control and Prevention) survey found that the percentage of dialysis units that had identified at least one patient with VRE increased from 11.5% in 1995 to 32.7% in 2000.(1) Atta et al. showed an incidence density of one case per 9.8 patient-years of follow-up in HD centers of East Baltimore(6), however, the incidence density in the present study was one case per 79.6 patient-months of follow-up. Although the incidence density of VRE colonization in the present study is substantially higher than that described in the former study(6), these may be due to different intervals between the performed cultures. 

The prospective design of the present study also allowed for a better evaluation of the patients for potential risk factors especially antibiotic, corticosteroid or immunosuppressive drug consumption and hospitalization during the study period. Previous studies have demonstrated an increased risk of VRE colonization with male gender(11), increased age(8), concomitant critical illnesses, severe underlying diseases and immunosuppression(10), anemia and leukocytosis(9), nonambulatory status(14), poor functional status(1,3), receipt of antibiotics(1,3,10), use of vancomycin(1,5,6,14), injection drug use(1,3) and hospitalization.(1,10,11) However, the present study only found association of antibiotic consumption with VRE colonization.

The relatively high numbers of VRE (22%) in our health institutions is probably due to inappropriate and high rate of administration of antibiotics, as in developing countries. Dan et al. demonstrated a low frequency (4.8%) of VRE colonization in hemodialysis patients due to low rate of antibiotic consumption and restrictions on vancomycin, used only upon approval by an infectious disease consultant.(10)

In conclusion, we can suggest that judicious use of antibiotics may be the most effective method of controlling the prevalence of VRE colonization in hemodialysis patients.
